# The Role of Transcriptional Factor Brachyury on Cell Cycle Regulation in Non-small Cell Lung Cancer

**DOI:** 10.3389/fonc.2020.01078

**Published:** 2020-07-03

**Authors:** Jingyi Xu, Ming Chen, Yinghui Wu, Hong Zhang, Jundong Zhou, Donglai Wang, Tianming Zou, Jun Shen

**Affiliations:** ^1^Department of Orthopeadic Surgery, The Affiliated Suzhou Hospital of Nanjing Medical University, Suzhou Municipal Hospital, Suzhou, China; ^2^Department of Orthopeadic Surgery, The Affiliated Wuxi No. 2 People's Hospital of Nanjing Medical University, Wuxi, China; ^3^Suzhou Cancer Center Core Laboratory, The Affiliated Suzhou Hospital of Nanjing Medical University, Suzhou Municipal Hospital, Suzhou, China

**Keywords:** brachyury, lung cancer, NSCLC, transcriptional factor, tumorigenesis

## Abstract

Lung cancer is the leading cause of cancer-related death, and non-small cell lung cancer (NSCLC) accounts for almost 80–85% of all lung cancer cases. The transcriptional factor brachyury has been verified to promote tumor cells migrate, invade, and metastasis in various types of tumors, whereas divergent roles of brachyury on cell proliferation have been reported in several types of tumor cells. In this study, we attempted to explore the effect of brachyury on the cell cycle progression and proliferation capability of NSCLC cells. Firstly, we performed RNA-sequence and ChIP-sequence to explore underlying downstream pathways regulated by brachyury. Cell proliferation and colony formation assays were utilized to detect the effect of brachyury on the proliferation ability of two types of lung NSCLC cells: H460 and Calu-1, which represent different brachyury expression levels. Following cell cycle and cell apoptosis assays were used to investigate the mechanism by which brachyury promotes NSCLC grow and progression. RNA-sequence and ChIP-sequence (ChIP-seq) showed that one of the vital downstream pathways regulated by brachyury involves in cell cycle progression. Through cell proliferation assays and colony formation assays, we found that inhibition of brachyury could decrease the capability of proliferation in H460 cells. We also found that brachyury overexpression could prevent the transition from G0/G1 to S phase in Calu-1 cells, and brachyury knockdown could decrease the transition of G2/M phase in H460 cells. The cell apoptosis assays showed that inhibition of brachyury could promote apoptosis in H460 cells. In this study we demonstrate that brachyury and downstream target genes together involve in tumor cell cycle regulation by inducing accelerated transition through G2/M, promote tumor cell proliferation and inhibit apoptosis in lung NSCLC H460 cells. Targeting brachyury expression could be developed into a promising avenue for the prevention of lung cancer progression.

## Introduction

Lung cancer is the leading cause of cancer-related death in both men and women, non-small cell lung cancer (NSCLC) accounts for almost 80–85% of all lung cancer cases (1). Histologically, NSCLC is mainly divided into adenocarcinoma, squamous cell carcinoma and large cell carcinoma. Different subtype has specific molecular and genomic signature that drives the progression and metastasis of tumor cells.

The human brachyury protein (Bry), the transcriptional factor regulating posterior mesoderm formation and notochord differentiation, has been reported as a specific and sensitive marker (2–5) and a master regulator of the oncogenic transcriptional network of chordoma (6). Further studies demonstrated up-regulation of *brachyury* gene occurs in various human tumors of epithelial origin, including lung, breast, colorectal, prostate cancer and others, but not in the majority of normal adult tissues (7–9). In primary lung carcinoma samples, brachyury mRNA expression was identified as a significant predictor in 5 year disease free survival and overall survival rate (10) and positively correlated with tumor stage and poor prognosis (8, 10, 11). Silencing of brachyury expression significantly diminished migratory, invasive and metastatic ability in endogenously positive lung cancer cells *in vitro* (8, 11), which suggests brachyury can be developed into a potential therapeutic target in anti-tumor treatment of lung cancer.

Several previous studies have demonstrated that brachyury drives epithelial-mesenchymal transition (EMT) in various types of human tumor cells, including lung carcinoma, breast carcinoma, among others, to promote progression and metastasis (8, 9, 12). In addition, as a master regulator, brachyury governs an elaborate oncogenic transcriptional network involving diverse signaling pathways (12), by one of which brachyury implicates in controlling cell cycle and regulating proliferation and apoptosis (8, 13, 14). Brachyury expression levels vary a lot among different subtypes of NSCLC tissue and cell lines, ranging from strong to almost no expression (15). Therefore, the role of brachyury in specific NSCLC subtype could be different and context-dependent.

Our previous study on the breast cancer cells (9) uncovered that brachyury promote tumor cell proliferate *in vitro* and *in vivo*. Whereas, another study by Palena et al. (16) demonstrated that silencing of brachyury in MDA-MB-436 cells significantly enhanced the proliferation ability of tumor cells, which means brachyury play an opposite role of what we reported. In regard to lung cancer cells, some studies demonstrated brachyury inhibits tumor cells grow and proliferate (8, 13). But recently Hu et al. (17) showed upregulated brachyury increases lung cancer cell growth and invasion. The cause of the divergent role played by brachyury on lung cancer cell proliferation still remains unclear. In this study, we attempted to explore the potential mechanism from the perspective of cell cycle regulation, which is one of the paramount downstream pathways regulated by brachyury.

## Materials and Methods

### Cell Lines and Cell Culture

Human large cell lung cancer cell line H460, Human lung cancer cell line Calu-1 were purchased from the State Key Laboratory of radiation medicine and radiation protection (Suzhou, China). H460 cells and Calu-1 cells were cultured in RPMI-1640 medium. The media (Gibco, Suzhou, China) were supplemented with 10% fetal bovine serum (FBS, Gibco, Australia) and 1% antibiotics (Penicillin-Streptomycin). Cells were incubated in a humidified atmosphere at 37°C and 5% CO_2_.

### RNA-Seq Transcriptome Analysis

Total RNA from MDA-MB-231 shNC/shBry was prepared and kept at 80°C. The RNA quality was determined using a Bioanalyzer 2200 (Agilent, Santa Clara, CA, USA). RNA with RIN (RNA integrity number)>8.0 was considered acceptable for cDNA library construction. Sequencing and bioinformatic analysis were performed by Shanghai Novelbio. Genes were considered to be significantly differentially expressed between groups when the *P* < 0.05 and the fold change of expression was more than 1.5.

### Chromatin Immunoprecipitation and Sequencing

To explore the underlying mechanisms of brachyury in lung cancer cells, Chromatin immunoprecipitation and sequencing (ChIP-seq) using wildtype MDA-MB-231 cells was performed. The ChIP assay kit (Millipore) was used to perform the ChIP assay. The anti-Bry antibodies used in this assay were purchased from R&D Systems (Bio-Techne, Minneapolis, MN). The Qubit® Fluorometer was used to determine the purity and concentration of DNA samples. TruSeq Nano DNA Sample Prep Kit (#FC-121–4002, Illumina, San Diego, CA) was used to end repair, tail and adaptor ligate DNA samples. AMPure XP beads were used to select the fragments of ~200–1,500 bp. The samples were diluted to a final concentration of 8 pM and cluster generation was then performed on the Illumina cBot using a HiSeq 3000/4000 PE Cluster Kit (#PE-410–1001, Illumina). Last, HiSeq 3000/4000 SBS Kit (300 cycles; #FC-410–1003, Illumina) was used to perform the sequencing on an Illumina HiSeq 4000. The data were then collected and analyzed.

### Construction of Cell Lines

To construct brachyury overexpression/knockdown cell lines, viral particles containing a small interfering RNA (siRNA-1 and siRNA-2) targeting brachyury or the human brachyury coding region purchased from GenePharma (Suzhou, China) were utilized in H460 cells and Calu-1 cells. The cell lines were constructed as described (9, 18) previously and validated using western blotting (siRNA-1: CGAATCCACATAGTGAGAGTT; siRNA-2: GAGGATGTTTCCGGTGCTGAA; siRNA-Control: TTCTCCGAACGTGTCACGT).

### Protein Extraction and Western Blotting

Total protein from lung cancer cells was extracted using a mammalian protein extraction reagent (MPER, Thermo Fisher Scientific, Waltham, MA, USA) containing a protease inhibitor cocktail (Thermo Fisher Scientific). An equivalent amount of protein was electrophoresed using 10% SDS gel electrophoresis and then transferred to the polyvinylidene fluoride (PVDF) membrane (Millipore, Billerica, MA, USA). The membranes were blocked with 5% skimmed milk for 1 h, and then incubated overnight with primary antibodies. The membranes were washed 3 times and incubated with secondary antibodies (Anti-Brachyury ab20680, Multi-sciences Biotechnology, Zhejiang, China). The membranes were then visualized using a chemiluminescence (ECL) (Multi-sciences Biotechnology) detection system. The primary antibodies used included anti-Brachyury (Abcam) and anti-GAPDH-HRP (MultiSciences, Zhejiang, China).

### Cell Proliferation Assay

Cell counting plates were used to detect cell proliferation after transfection. 3 × 10^3^ Calu-1 and H460 cells were seeded in 24-well plates and allowed to adhere. Cell viability was measured every day for 4 days using Cell Counting Kit-8 (Dojindo Laboratories, Kumamoto, Japan) according to the manufacturer's instructions. All results were recorded and the cell proliferation curves were drawn.

### Colony Formation Assay

1 × 10^2^ Calu-1 (Calu-1 Bry cells and NC cells) and H460 cells (H460 siBry-1 cells, siBry-2 cells and siNC cells) were used to perform the colony formation assay. Cells were cultured in the RPMI-1640 medium containing 10% fetal bovine serum and 1% antibiotics for 10 days. The remaining colonies were stained with crystal violet and then recorded.

### Cell Cycle Assay

Cells (2 × 10^5^) were harvested at 24 h after siRNA transfection, and fixed with cold 70% alcohol at-20°C overnight. Alcohol was removed and cells were washed twice with cold phosphate buffer saline (PBS). Cells were stained with propidium iodide solution containing 20 μg/ml RNase and incubated at room temperature for 30 min. After filtering by a nylon mesh filter, cell cycle was performed on a fluorescence-activated cell sorting (FACS, FACSVerse) analysis. Data were analyzed using the Flowjo software (Version 7.6.1, Tree Star Software, San Carlos, CA, USA).

### Cell Apoptosis Assay

#### Sub-G1 Method

Calu-1 and H460 cells were first seeded in 6-well plates. 2 × 10^5^ cells cultured for 24 h were harvested from each well and fixed with 70% alcohol in refrigerator. The alcohol were washed off with PBS, and the cells were resuspended in 1 ml DNA staining solution at room temperature for 30 min in the dark. Flow cytometry was used to select blue excitation light with a wavelength of 488 nm, and simultaneously measure red fluorescence and forward-angle scattered light.

#### Annexin V/PI Method

Cells were harvested at 24 h after transfection, and stained with Annexin V-FITC and propidium iodide (Annexin V-FITC apoptosis detection kit, B.D. Biosciences Pharmingen, San Jose, CA, USA). Then cells were put in the dark for 15 min at room temperature. The apoptosis rate was detected by BD FACS Calibur (Beckman Coulter, CA, U.S.A.).

### Statistical Analysis

Values as shown were mean ± S.E.M. of at least three independent repeats. Statistical analysis was performed using SPSS17.0 software (IBM Corp, Armonk, NY). Student's *t*-test was used to compare the differences between two groups. The difference between more than two groups was analyzed by single factor analysis of variance (ANOVA). *P* < 0.05 was defined as statistically significant.

## Results

### Differentially Expressed Genes in Brachyury-Knocking Down MDA-MB-231 Cells and Control Cells

**Figure 1:**
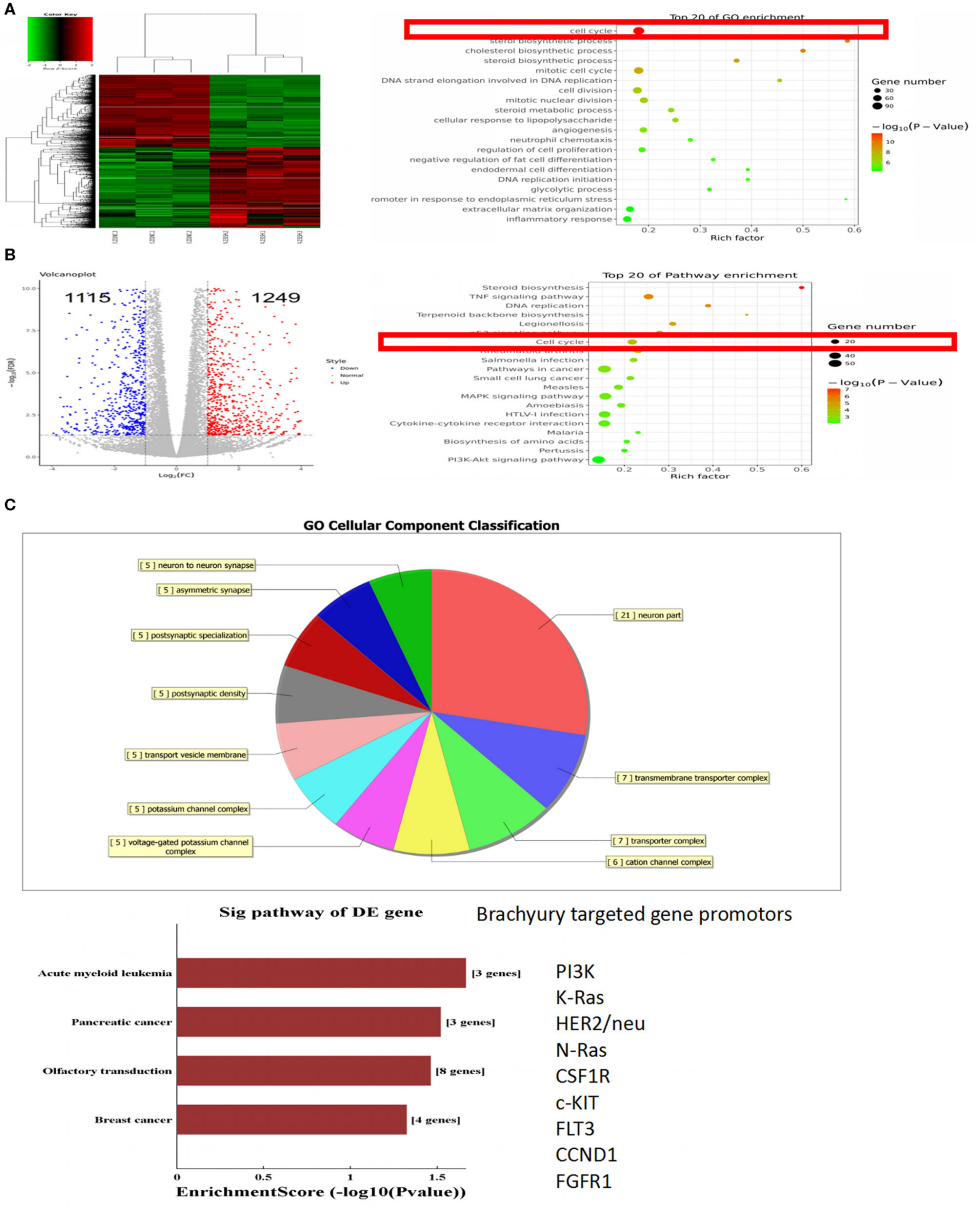
RNA-sequence and ChIP-sequence analyses of MDA-MB-231 cells. **(A)** Heat map of differentially expressed transcripts, Gene ontology (GO) analysis, and **(B)** pathway analysis, based on all identified transcripts. **(C)** Cellular component classification analysis and targeted genes of ChIP-seq. *P*-value of GO analysis and enrichment of pathway analysis are listed for each category. *P*-value of pathway is colored in red (*P* < 0.05).

Brachyury was constitutively overexpressed in breast cancer MDA-MB-231 cells. To explore the potential downstream targets and pathways regulated by brachyury in tumor cells, RNA-Sequence was performed to profile the transcriptome in brachyury-knockdown MDA-MB-231 cells (MDA-MB-231 shBry) vs. control MDA-MB-231 cells. A total of 2,364 genes were identified to be differentially expressed, which were further analyzed to characterize potential pathways or biological processes. Involving pathways mainly includes: Steroid biosynthesis, TNF signaling pathway, and DNA replication, etc. The gene ontology analysis revealed some biological processes are involved: cell cycle, sterol biosynthetic process and the cholesterol biosynthetic process, etc. (Figures 1A,B). For ChIP-seq assays, the results showed that the cellular components were classified into 11 types, including neuron, asymmetric synapse, postsynaptic specialization, etc. Furthermore, ChIP-seq results in this study showed that several downstream genes were significantly associated with brachyury-binding events, including PIK3, K-RAS, HER2, N-Ras, CSF1R, etc. PIK3 and K-RAS are potential target genes of brachyury according to *P*-value (Figure 1C), both of which are involved in cell cycle regulation pathway. Combining and integrating RNA-seq and ChIP-seq results, we speculated that brachyury promotes tumor cell grow and progress through regulation of cell cycle. The predictive target genes above in breast cancer cells were further verified to be expressed highly in endogenous brachyury-expressing lung cancer cells using PCR assays.

### Brachyury Knockdown Decreased Lung Cancer Cell Proliferation *in vitro*

**Figure 2:**
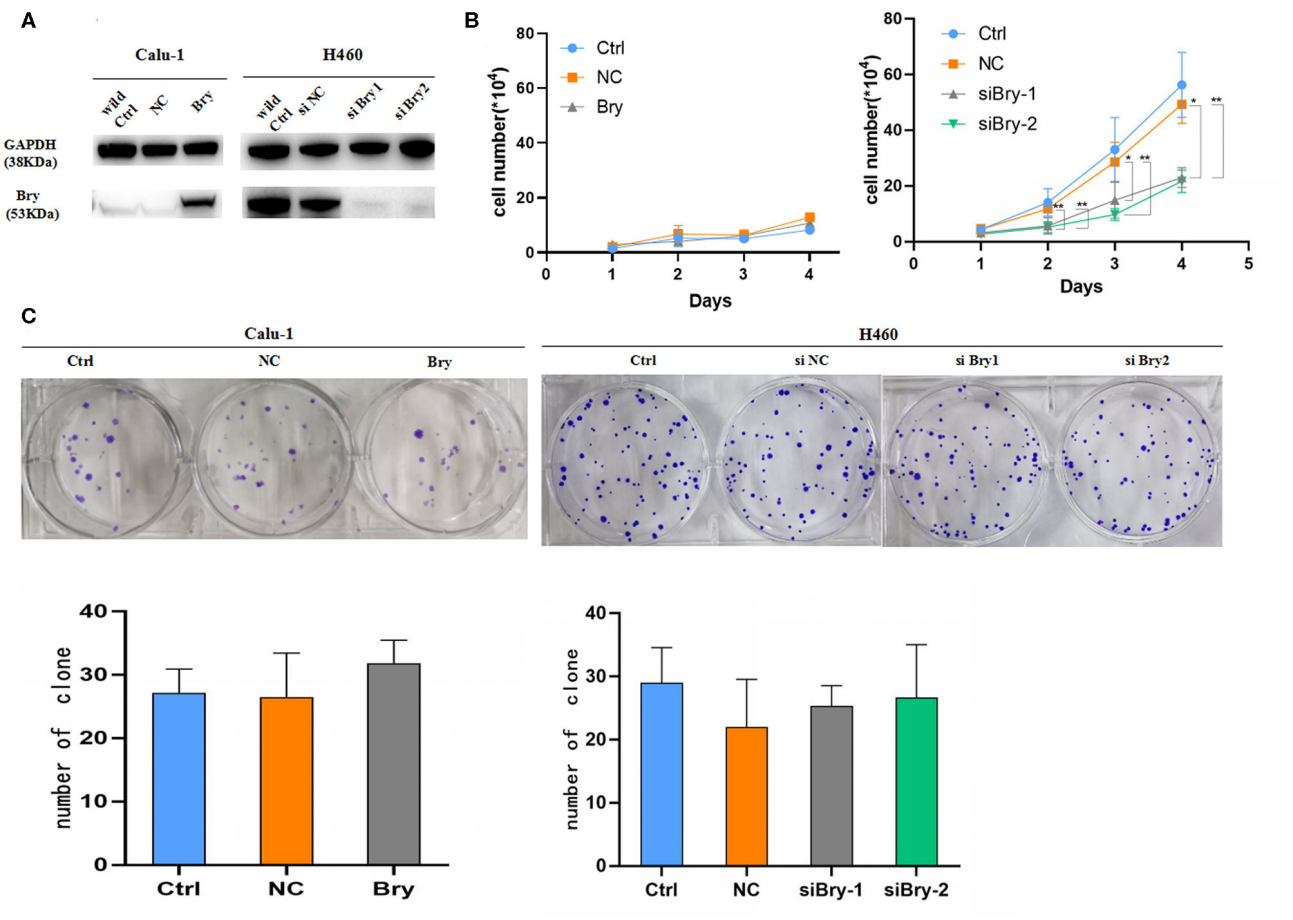
Proliferation assays and colony formation assays to assess the effect of Bry in lung cancer cells. **(A)** The protein levels of Bry in Bry-overexpression Calu-1 cells or Bry-knockdown H460 cells compared with their respective control cells. **(B)** Cell proliferation assays to assess the proliferation capacity of Bry-overexpression Calu-1 cells and Bry-knockdown H460 cells. **(C)** Colony formation assays to assess the proliferation capacity of Bry-overexpression Calu-1 cells and Bry-knockdown H460 cells (*P < 0.05, **P < 0.01).

To identify the role of brachyury on the proliferation capability of lung NSCLC cells *in vitro*, Calu-1 cell line (absence of brachyury expression) and H460 cell line (endogenously high expression of brachyury) were utilized for further study. Inhibition of brachyury in H460 cells (H460 siBry cells) and brachyury overexpression in Calu-1(Calu-1 Bry cells) were used to investigate whether brachyury could enhance the proliferation capability of lung cancer cells (Figure 2A). The results showed that brachyury overexpression in Calu-1 cells had no significant effect on the proliferation, while brachyury knockdown in H460 cells reduced the proliferation ability compared to the control cells (Figure 2B). Brachyury overexpression or knockdown had no effect on the ability of colony formation in Calu-1 and H460 cells (Figure 2C). In summary, brachyury knockdown could decrease the proliferation capability in H460 cells.

### The Effect of Brachyury Expression on Cell Cycle

**Figure 3:**
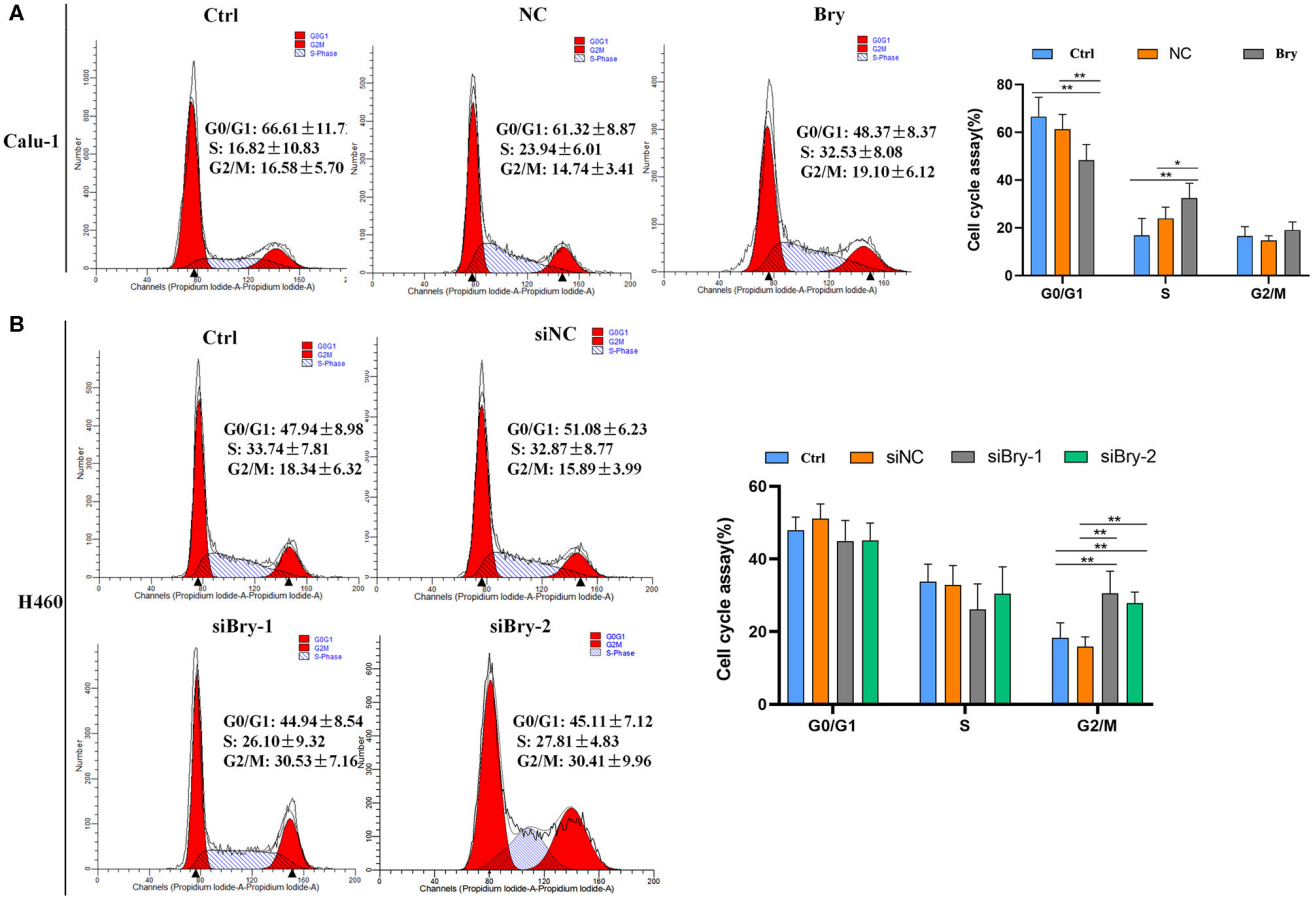
The effect of Bry on cell cycle. **(A)** Cell cycle analysis of Bry-overexpression Calu-1 cells. Overexpression of brachyury in Calu-1 cells could promote the transition from G0/G1 to S phase. **(B)** Cell cycle analysis of Bry-knockdown H460 cells. Inhibition of brachyury expression in H460 cells could increase the percentage of G2/M phase (*P < 0.05, **P < 0.01).

The impact of brachyury on cell cycle progression was also investigated. The cell cycle experiment was performed and analyzed by flow cytometry. Calu-1 Bry cells demonstrated significant lower G0/G1 fraction (48.37%, Figure 3A) than control cells (61.32%), and the percentage of S phase in Calu-1 Bry cells (32.53%, Figure 3A) was markedly higher than control cells (23.94%, Figure 3A). However, the percentage of G2/M phase did not have significant difference (Figure 3A). Hence, overexpression of brachyury in Calu-1 cells could inhibit the transition from G0/G1 to S phase.

For H460 cells, the percentage of G0/G1 and S phase did not have significant difference between the brachyury knockdown group and the control group (Figure 3B). However, the percentages of G2/M in the H460 siBry-1 and H460 siBry-2 (26.10% and 27.81%, respective, Figure 3B) were significantly higher than the control cells (15.89%, Figure 3B). We concluded that inhibition of brachyury expression in H460 cells could prevent G2/M transition of cell cycle progression.

### Brachyury Knockdown Promotes Apoptosis in H460 Cells

**Figure 4:**
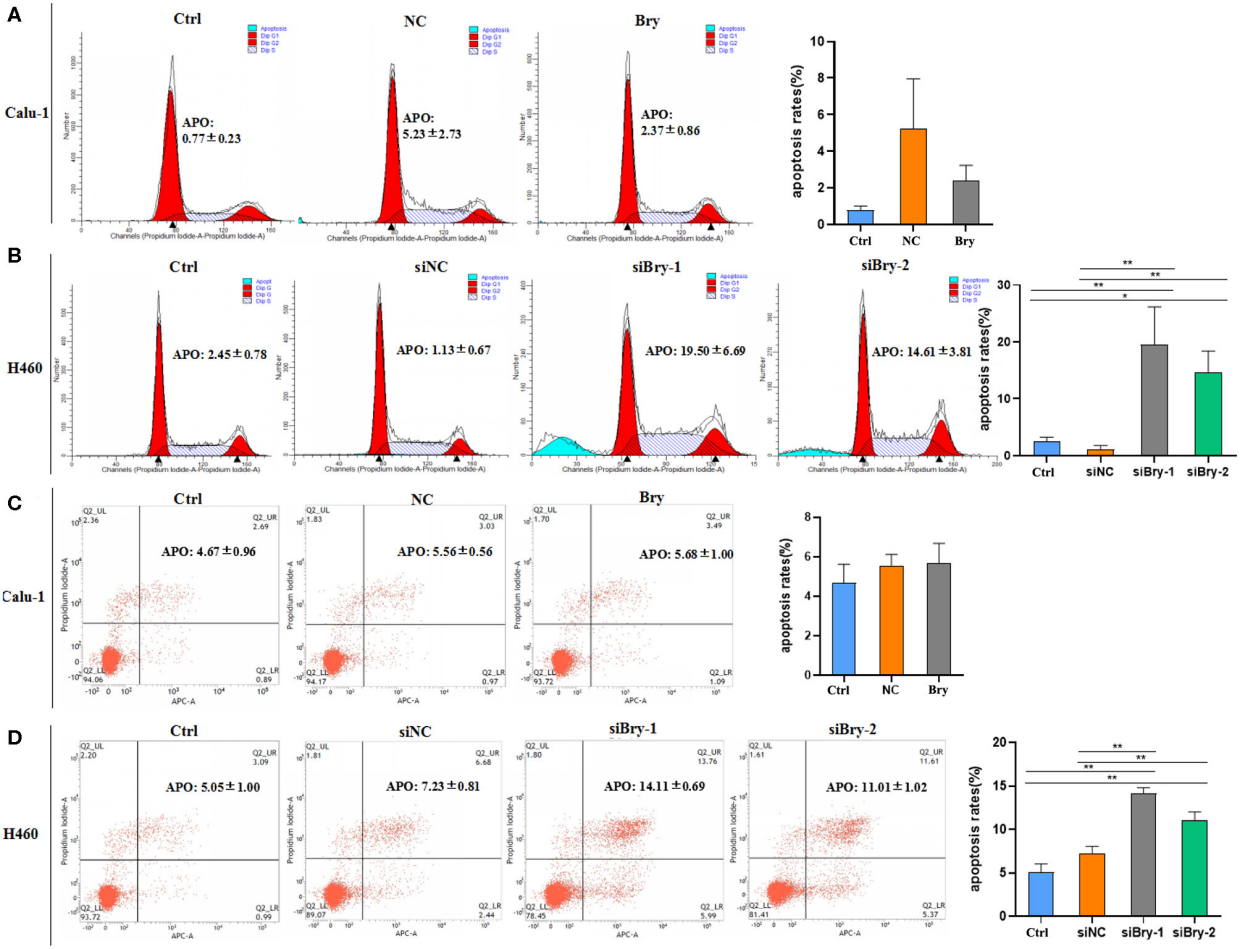
The effect of Bry on cell apoptosis. **(A)** The effect of Bry on apoptosis of Bry-overexpression Calu-1 cells. There was no significant difference between the experimental group and the control group. **(B)** The effect of Bry on apoptosis of Bry-knockdown H460 cells. **(C)** Annexin V/PI method to analyze the effect of Bry on apoptosis of Bry-overexpression Calu-1 cells. There was no significant difference between the experimental group and the control group. **(D)** Annexin V/PI method to analyze of the effect of Bry on apoptosis of Bry-knockdown H460 cells (*P < 0.05, **P < 0.01).

Next, we investigated the effect of brachyury on cell apoptosis in H460 cell line and Calu-1 cell line. After overexpression of brachyury in Calu-1 cells, the percentage of apoptotic cells was assessed using sub-G1 and Annexin V/ PI method, followed by flow cytometry (Figures 4A,C). The results showed that the percentage of apoptotic Calu-1 Bry cells (2.37%) was not significant lower than control cells (5.23%), and a dot-plot of Annexin V-FITC fluorescence vs. PI fluorescence indicated a non-significant increase in the percentage of apoptotic cells after overexpression of brachyury (Figure 4C). Similarly, we evaluated the apoptosis of H460 cells after knockdown with siRNA-1 and siRNA-2. The results showed that the percentage of apoptotic H460 siBry-1 cells (19.50%) and apoptotic H460 siBry-2 cells (14.61%) was significant higher than the control cells (1.13%) (Figure 4B). Figure 4D was consistent with Figure 4B. In conclusion, brachyury knockdown could expedite apoptosis of H460 cells.

## Discussion

A comparable range of brachyury mRNA expression levels has been observed in the lung tumor tissue samples and the various lung carcinoma cell lines, which suggests available cell line models can be used as useful tools to ravel the individual mechanism associated with tumorigenesis and progression (15). Elevated expression of brachyury in H460 cells has been validated to associate with invasive and metastasis capability of tumor cells along with survival benefit (8, 13). However, human squamous cell carcinoma cell line Calu-1 was reported and characterized by negative brachyury expression (15). It is because that these two types of cell lines represent different subtype of NSCLC. So we utilized Calu-1cells as the control to reveal the specific role of brachyury in endogenously brachyury-expressing lung tumor cell. Compared with control cells, inhibition of brachyury expression in H460 cells demonstrated significantly diminished proliferation capability. However, overexpression of brachyury did not result in significantly enhanced proliferation of Calu-1 cells. In the present study, we found that brachyury plays a role of promoting cell proliferation in lung H460 cells, which is consistent with our previous study of brachyury on breast cancer cells (9) and other reports on chordoma (3, 6, 19, 20), prostate cancer (21), colorectal cancer (22),adenoid cystic carcinoma (13), among others (20, 21).

H460 cells with silencing of brachyury (H460 siBry-1, H460 siBry-2) demonstrated significantly higher G2/M fraction, and insignificantly decreased G1/G0 and S fraction than control with highly endogenous expression of brachyury. In addition, brachyury-silenced H460 cells demonstrated significantly increased apoptosis. Altogether, these results indicate that brachyury inhibits apoptosis in H460 cells, although over-expression of brachyury did not demonstrate the similar anti-apoptosis effect in Calu-1 cells. The reason we speculate is that the downstream brachyury-responsive elements are in the state of inactivation or silencing due to the absence of endogenous brachyury stimulation in Calu-1 cells. The exogenously forced brachyury expression cannot find and bind with corresponding functioning targets.

Based on the gene expression microarray data and ChIP-seq data, gene ontology (GO) functional enrichment analysis of differentially expressed genes (DEGs) between wild brachyury-expressing and absence of expression breast cancer cells was performed to uncover the downstream target genes of brachyury. The up-regulated DEGs are listed in Figure 1C. Further GO annotation indicates that the top two PI3K and K-Ras are mainly involved in the regulation of cell cycle and proliferation. Following verified experiments were performed to confirm that these top DEGs were expressed samely in endogenously brachyury-expressing lung cancer cells.

K-Ras is one member of the *ras* gene family, which encodes small GTP-binding proteins. Five- to 50-fold amplification of the wild-type gene or mutation can make it convert into oncogene (23). Activated Ras oncogenes have been found in a variety of human tumors (24), and K-Ras has been considered as the most commonly altered oncogene in NSCLC, especially in adenocarcinoma (25, 26). PI3K is one of three major downstream effectors of K-Ras, and its activation initiates a signal transduction cascade that promotes cancer cell growth, survival and metabolism (27). In addition, RAS–PI3K pathway has been also reported to mediate the anti-apoptotic function of oncogenic RAS (28) and regulate the expression or activity of apoptotic-relating molecules or proteins (29).

In this study we demonstrate that brachyury and downstream target genes together involve in tumor cell cycle regulation by inducing accelerated transition through G2/M, promote tumor cell proliferation and inhibit apoptosis in lung NSCLC H460 cells. Targeting brachyury and downstream effectors pathway has the potential to be developed into a promising treatment strategy, which has far reaching significance for the therapy of NSCLC.

## Data Availability Statement

The datasets presented in this study can be found in online repositories. The names of the repository/repositories and accession number(s) can be found below: NCBI Gene Expression Omnibus (GSE151170, GSE151171, GSE151172).

## Author Contributions

JX carried out cell culture-based assays. MC carried out RNA-Seq transcriptome analysis and ChIP-sequence assay. YW performed data statistical analysis and wrote the manuscript. HZ, JZ, DW, and TZ carried out data statistical analysis. JS designed, supervised the study, and wrote the manuscript. All authors contributed to the article and approved the submitted version.

## Conflict of Interest

The authors declare that the research was conducted in the absence of any commercial or financial relationships that could be construed as a potential conflict of interest.
